# Blind Self-Supervised Denoising of In Situ BOTDR Strain Data Using TrendBlend-BSFormer for Underwater Flexible Mattress Monitoring

**DOI:** 10.3390/s26123663

**Published:** 2026-06-08

**Authors:** Jing Liu, Pengfei Jin, Zhixuan Zhang, Xianglong Wei

**Affiliations:** 1 The National Key Laboratory of Water Disaster Prevention, Nanjing Hydraulic Research Institute, Nanjing 210029, China; 2 Shanghai Water Conservancy Management Center, Shanghai 200002, China; 3 State Key Laboratory of Water Cycle and Water Security, Hohai University, Nanjing 210024, China

**Keywords:** distributed fiber optic sensing, BOTDR, blind self-supervised learning, denoising, flexible mattress, strain monitoring

## Abstract

The long-term stability of submerged sandbars and protected shorelines in large alluvial rivers depends on the serviceability of flexible mattresses installed on the riverbed. Distributed fiber optic sensing is one of the few practical methods for monitoring deformation along these underwater systems over engineering-scale distances. Yet BOTDR-derived strain-difference profiles are often heavily contaminated by noise and rarely have reliable clean references. To address this issue, this study develops TrendBlend-BSFormer, a blind self-supervised denoising framework for in situ BOTDR strain data from underwater flexible mattresses. The framework combines four key features: blind-spot masking, a one-dimensional encoder decoder backbone, a Transformer bottleneck for long-range spatial dependence, and a multi-scale trend-detail blending branch with dual signal-noise heads. The framework was validated using annual and daily BOTDR field data from the Yudaizhou shoreline protection project in the Yangtze River, containing 9343 and 9875 valid measurement points, respectively. TrendBlend-BSFormer achieved pseudo-SNR/RMSE/MAE values of 14.22 dB, 15.03 με and 12.05 με for the annual data set and 5.32 dB, 8.02 με and 6.45 με for the daily data set, improving the pseudo-SNR by 1.45 dB and 2.95 dB relative to the published BiLSTM-CNN benchmark. It also reduced the high-frequency energy ratio from 0.172 to 0.011 for the annual data and from 0.424 to 0.112 for the daily data. The denoised profiles suppress isolated spikes while preserving mechanically plausible peaks, valleys, and short-range fluctuations, indicating that blind self-supervised denoising can provide a more physically credible strategy for BOTDR-based monitoring in complex underwater environments.

## 1. Introduction

**Figure 1:**
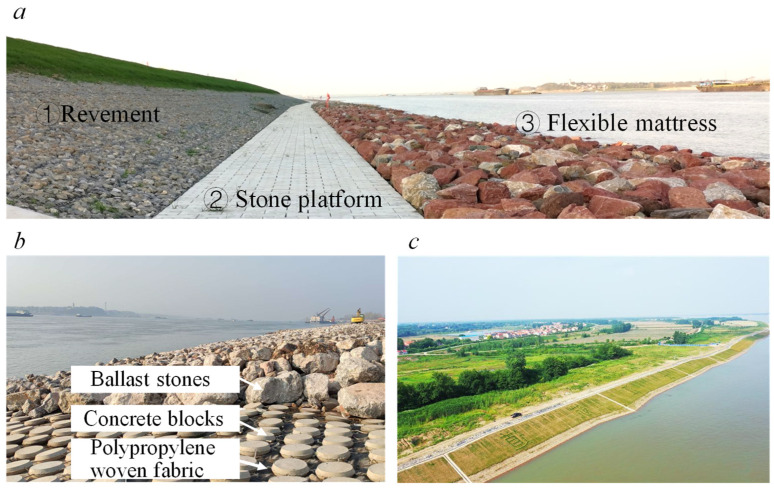
Flexible mattress for bankline protection: (**a**) the entire protection works; (**b**) components of the flexible mattress; (**c**) overall view of the completed works.

The stability of submerged sandbars is critical to the serviceability of navigation channels, shoreline-protection systems, and other river-training works in large alluvial rivers. In the Yangtze River, flexible mattresses (Figure 1) are widely employed to protect shoals, revetments, and submerged foundations against scour and erosion because they can accommodate bed deformation while maintaining continuous coverage of the riverbed surface [1]. Once installed underwater, however, these mattresses become spatially extensive, deformable, and difficult-to-access structures whose condition cannot be adequately characterized by sparse displacement measurements or periodic topographic surveys alone. Distributed fiber optic sensing offers an effective alternative by enabling continuous deformation monitoring along submerged flexible mattresses [2]. These strain responses are particularly informative because they reflect the adjustment of the protected riverbed to flow forcing, sediment redistribution, settlement, and local instability. Among the available techniques, Brillouin-scattering-based distributed sensing is especially attractive because of its long sensing range, dense spatial coverage, and robustness under harsh environmental conditions. Recent reviews have highlighted its growing importance in structural, geotechnical, tunnel, and geohazard monitoring, while also emphasizing that its engineering value depends not only on sensor deployment but also on the reliability and interpretability of the measured strain data [3,4,5,6,7,8]. Beyond fiber-based strain sensing, underwater optical monitoring has also benefited from adaptive optical strategies, including adaptive exposure optimization for underwater optical camera communication, adaptive-optics correction using bimorph mirrors, and adaptive-optic underwater imaging [9,10,11]. These studies show that underwater optical observations often require active or computational compensation for scattering, turbulence, attenuation, and intensity fluctuation. However, such approaches primarily correct free-space optical images, beams, or communication signals, whereas BOTDR-based flexible-mattress monitoring requires denoising of one-dimensional distributed strain sequences measured along a submerged cable. This distinction motivates the development of a denoising framework tailored to BOTDR strain data.

Despite this promise, the engineering utility of BOTDR measurements for underwater flexible-mattress systems remains limited by a practical challenge: data interpretation. Brillouin-based strain measurements are inherently susceptible to uncertainty arising from weak spontaneous scattering, environmental disturbances, and site-specific noise contamination. As a result, the measured strain-difference curves often contain high-frequency fluctuations and isolated spikes that obscure mechanically meaningful responses [12,13,14]. Conventional post-processing methods, including wavelet thresholding and Kalman filtering, remain attractive because of their simplicity and interpretability; however, their effectiveness is often highly sensitive to parameter selection, and stronger spike suppression is frequently achieved at the cost of local detail preservation [15,16]. At the same time, machine learning has assumed an increasingly important role in optical-fiber sensing workflows, including Brillouin data processing and broader distributed sensing applications [17,18]. Blind and weakly supervised denoising strategies have likewise advanced rapidly, from Noise2Noise, Noise2Self, and Noise2Void to more recent self-supervised methods developed for distributed acoustic sensing data [2,19,20,21,22,23]. For BOTDR data acquired from underwater flexible mattresses, however, denoising remains particularly challenging because only noisy field measurements are available, clean labels are absent, and the denoised results must be credible not only in quantitative terms but also in visual form for engineering interpretation. Therefore, further methodological improvement is still required to better balance noise suppression, preservation of local peaks and valleys, and the overall naturalness of the denoised strain curves.

Against this background, this study develops TrendBlend-BSFormer, a blind self-supervised denoising framework for BOTDR strain-difference data measured on underwater flexible mattresses. The proposed method establishes a trend-aware denoising architecture that integrates blind-spot self-supervision, long-range spatial-context modeling, and multi-scale trend-detail blending, thereby suppressing noise without oversmoothing the strain profile and preserving its in situ engineering interpretability. The remainder of this paper is organized as follows. Section 2 introduces the field data source and monitoring background. Section 3 presents the proposed framework, comparison methods, and evaluation metrics. Section 4 reports and discusses the results. Section 5 summarizes the main conclusions.

## 2. Measured Strain Data Sets

**Figure 2:**
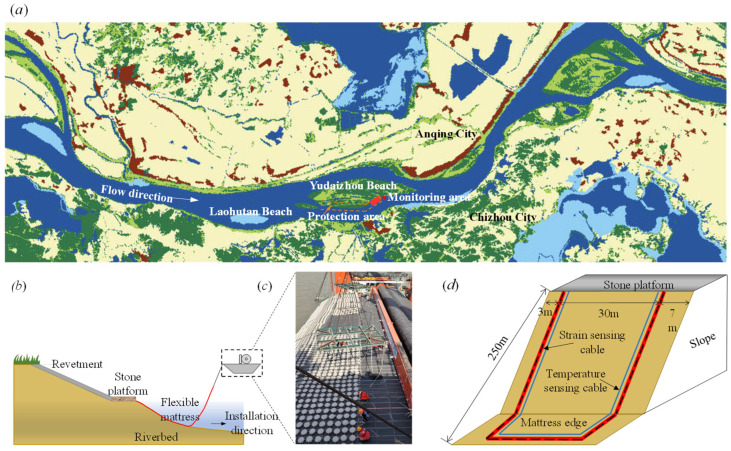
(**a**) layout of research area; (**b**,**c**) construction process of flexible mattress; (**d**) layout the sensing cables and flexible displacement meter on the surface of flexible mattress [2].

The field data were obtained from the Yudaizhou project in the Dongliu Reach of the lower Yangtze River (Figure 2), where a distributed optical-fiber monitoring system was installed on seven flexible mattresses located at the downstream end of the protected reach, namely YD1 and YD5-YD10. The monitoring project, cable deployment scheme, and engineering background of the Yudaizhou shoreline protection system were described in detail by Wei et al. [2]; only the information directly relevant to the present denoising study is summarized here. In this system, armored sensing cables were installed synchronously with mattress construction, arranged along the mattress perimeter, and interrogated using single-ended BOTDR. This measurement configuration was adopted because reliable access to both cable ends is difficult to maintain in an underwater flexible-mattress environment. Installation of the monitoring system was completed in January 2020, and ballast-stone placement was completed in June 2020. Among the monitored mattresses, YD9 was selected here as the representative section because it preserved a continuous U-shaped sensing path, whereas the adjacent YD10 section experienced cable breakage. The strain sequences analyzed in this study were extracted from a 450 m monitored segment associated with YD9.

**Figure 3:**
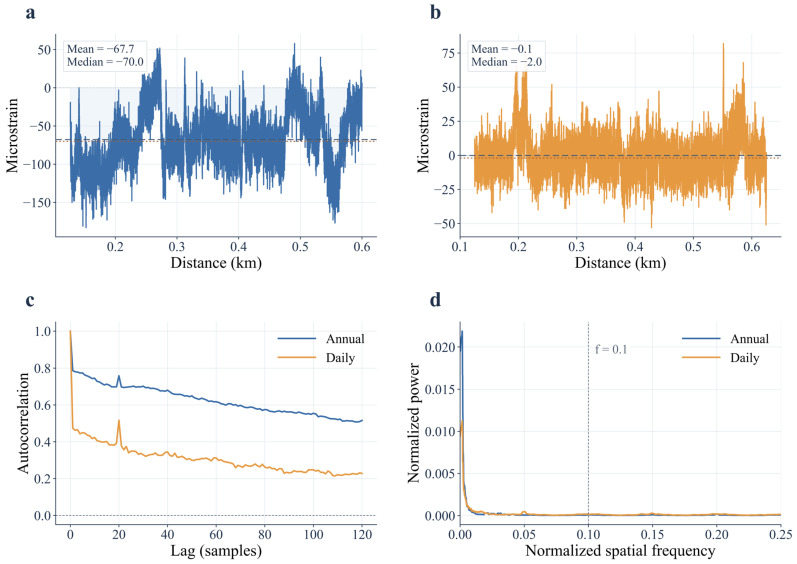
Statistical characterization of the annual and daily BOTDR strain-difference data sets: (**a**) annual raw strain-difference profile with mean and median lines; (**b**) daily raw strain-difference profile with mean and median lines; (**c**) short-range spatial autocorrelation decay of the annual and daily data; (**d**) normalized spatial spectral density of the annual and daily data.

Two BOTDR strain-difference data sets were constructed from this section. The annual data set was defined as the strain difference between 18 November 2020 and 28 November 2021, whereas the daily data set was defined as the strain difference between 18 November 2020 and 19 November 2020. To improve clarity, the principal numerical information that was previously described in the text is reorganized in Table 1, including the number of valid measurement points, amplitude statistics, robust descriptors, short-range dependence, and spectral descriptors. The corresponding profiles and structure-sensitive curves are plotted in Figure 3. The annual sequence is more strongly shifted away from zero and is dominated by broader, more persistent spatial variation. By contrast, the daily sequence remains closer to zero in mean level but has stronger asymmetry and richer high-frequency content, as shown by its higher skewness, excess kurtosis, spectral entropy, and high-frequency energy ratio. A small runout, or shoulder, is visible around lag 20 in the autocorrelation curves in Figure 3c. Since the reconstructed distance coordinate has an approximate sampling interval of 0.05 m, this lag corresponds to about 1 m along the cable. The runout is therefore interpreted as a scale-dependent autocorrelation shoulder associated with effective spatial averaging, local cable-mattress contact, and non-stationary strain patches, rather than as evidence of a separate periodic deformation mode. This interpretation is also supported by the absence of a narrow isolated peak at the corresponding frequency in the normalized spatial spectra.

## 3. Denoising Method for Measured Data

### 3.1. Proposed Method: TrendBlend-BSFormer

The proposed method was motivated by two key characteristics of the in situ BOTDR strain-difference data acquired from underwater flexible mattresses. First, these sequences are observed only in contaminated form, so an effective denoising model must be trained directly on noisy observations rather than on clean labels or externally constructed references. Under such conditions, methods relying solely on local regression or conventional sequence smoothing are prone to two opposing failure modes: they may either remain overly faithful to the raw noisy measurements or oversuppress fluctuations to the extent that mechanically meaningful local responses are attenuated. Second, the annual and daily data exhibit markedly different dominant scales. Annual strain differences are characterized primarily by broad and persistent spatial structures, whereas daily differences contain denser short-wavelength oscillations and stronger high-frequency variability. Consequently, a robust denoising strategy should explicitly distinguish trend-like components from fine-scale details instead of treating the entire sequence as a homogeneous signal class. From this perspective, TrendBlend-BSFormer was developed as a unified one-dimensional denoising framework that integrates blind self-supervised learning, multi-scale trend-detail blending, and a Transformer-augmented encoder–decoder backbone.

#### 3.1.1. Network Architecture

**Figure 4:**
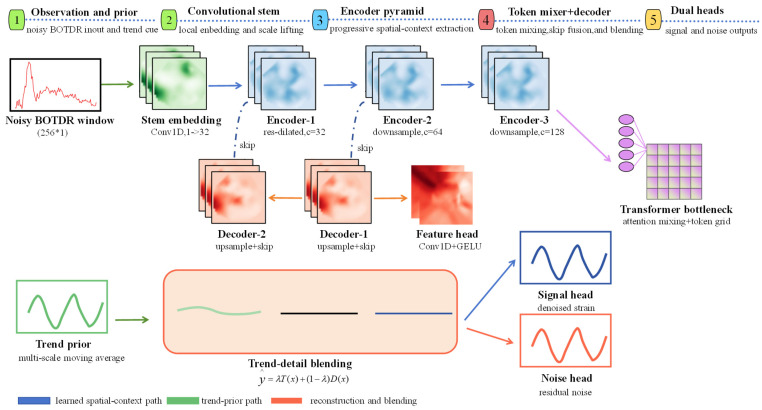
Overall architecture of TrendBlend-BSFormer: multi-scale trend prior, encoder–decoder backbone, Transformer bottleneck, skip-connected decoder, and dual signal-noise heads.

The overall architecture is summarized in Figure 4. The framework couples a multi-scale trend-prior branch with a shared encoder–decoder backbone, a Transformer bottleneck, and dual signal-noise reconstruction heads. For each input window, the backbone begins with a Conv1D stem that maps the raw strain sequence from one channel to 32 channels, followed by a 1D U-Net-like encoder–decoder. The encoder–decoder follows the U-Net design logic, whereas the bottleneck uses self-attention to capture long-range dependencies [21,22].

The decoder mirrors the encoder through transposed convolutions and skip connections, allowing the model to recover local details after global-context aggregation. A shared feature head feeds two output branches: a signal head that estimates the denoised strain and a noise head that estimates the residual noise. In contrast to a purely implicit regression framework, the dual-head design imposes a weak source-separation prior by encouraging the model to represent the measured input as the sum of a denoised component and a residual-noise component.

To accommodate the cross-scale deformation mechanisms of submerged engineering structures under complex hydrodynamic environments, a parallel multi-scale trend-prior branch is coupled with the deep learned backbone. For an in situ underwater flexible mattress, its measured strain-difference profile typically superimposes macro-scale continuous geo-structural displacement (driven by cumulative riverbed evolution and multi-period settlement) and micro-scale localized structural perturbations (induced by uneven ballast distribution or local boundary scour). Formally, let $x=[x_{1},x_{2},\ldots ,x_{t}]\in R^{1\times T}$ denote one standardized BOTDR strain-difference input window, and ${{MA}_{k}(x)}_{i}$ denote the local moving average centered at position *i* with an odd kernel size *k* under reflected boundary padding. The baseline macro-morphology is initialized via a multi-scale convex blend as formulated in Equation (1):

(1) $$T_{0}(x)=0.25{MA}_{k_{s}}(x)+0.45{MA}_{k_{m}}(x)+0.30{MA}_{k_{l}}(x)$$

To faithfully adapt to the dominant riverbed evolution scales, the annual model implements an expansive kernel hierarchy (7/17/33 points, equivalent to a spatial domain of 0.35 m/0.85 m/1.65 m) to shield the continuous seasonal-scale geo-structural deformation trends from point-wise noise corruption. Conversely, the daily model utilizes tightly bounded kernels (5/13/25 points) to stay highly responsive to high-frequency short-wavelength structural adjustments under immediate flow forcing without inducing oversmoothing artifacts.

The learning-based signal head within the 1D U-Net backbone predicts a bounded structural correction term $C\left(x\right)=\gamma tanh(g_{\theta }(x))$, where $g_{\theta }(x)$ is the raw feature mapping and γ governs the maximum allowable envelope for macro-trend adjustment. The final adaptive trend candidate is thus defined by Equation (2):

(2) $$T(x)=T_{0}(x)+\gamma tanh(C(x))$$

Concurrently, a micro-detail preserving candidate is formulated by injecting the localized features back into the raw observation sequence as defined in Equation (3):

(3) D(x)=x+ηtanh(C(x))

where *η* serves as the hyperparameter modulating detail injection. The final denoised output *y* is synthesized as an adaptive convex combination of the decoupled structural trend and the localized details in Equation (4):

(4) $$\hat{y}=\lambda T(x)+(1-\lambda )D(x)$$

where *λ* represents the trend-blend ratio regulating the macro-to-micro structural balance. Finally, to complement the weak source-separation prior, the residual-noise sequence *n* is extracted via Equation (5):

(5) $$\hat{n}=x-\hat{y}+0.1N(x)$$

with *N*(*x*) denoting the auxiliary output from the noise-head branch. In our final validation configuration, the structural hyperparameters are frozen at *λ* = 0.35, *γ* = 1.4, and *η* = 0.9, striking an optimal, interpretable trade-off between strict mathematical consistency and physically plausible curve morphology.

#### 3.1.2. Blind Self-Supervised Training Strategy

**Figure 5:**
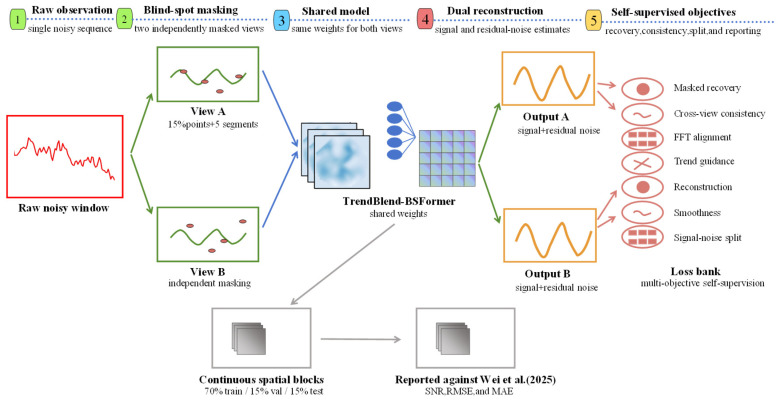
Blind self-supervised training workflow of TrendBlend-BSFormer. The reported evaluation metrics, including SNR, RMSE, and MAE, are benchmarked against Wei et al. (2025) [2].

The training strategy follows the blind-spot self-supervision principle rather than supervised fitting to unavailable clean labels. For each input window, two independently masked views are created. In each view, 15% of positions are randomly selected for point masking, and five additional contiguous segments of length 3–7 are masked. Masked positions are replaced by local linear interpolation plus small random perturbations derived from local variance, which prevents the network from trivially learning the identity map. Because the target is still the original noisy signal, the network is only penalized at the masked positions, which enforces a J-invariant learning mechanism similar in spirit to Noise2Self and Noise2Void [20,21]. The blind self-supervised training strategy is summarized in Figure 5.

Two independently masked views are processed by a shared model, and optimization is jointly guided by masked reconstruction, inter-view consistency, frequency-domain stability, trend alignment, smoothness regularization, and signal-noise separation constraints.

The total training loss is defined by Equation (6):

(6) $$L=L_{mask}+\alpha L_{cons}+\beta L_{rec}+\delta L_{smooth}+\phi L_{freq}+\mu L_{trend}+\nu L_{split},$$

where *L_mask_* is the masked SmoothL1 reconstruction loss, *L_cons_* is the consistency loss between the two masked views, *L_rec_* enforces *x* approximately equal to the sum of the denoised component $\hat{y}$ and the residual-noise component $\hat{n}$, *L_smooth_* is an edge-aware second-difference regularizer, *L_freq_* is a multi-resolution FFT consistency loss, *L_trend_* aligns the denoised output with a multi-scale pseudo-trend, and *L_spli__t_* penalizes leakage of high-frequency components into the denoised signal and low-frequency components into the residual-noise branch. This loss design differs from the BiLSTM-CNN framework [2] in two aspects. First, it does not depend on one-step sequence prediction as a surrogate denoising target. Second, it explicitly constrains the denoised output to be smooth in non-edge regions while preserving local fluctuations through the trend-detail blending branch.

#### 3.1.3. Training Details

The model was implemented in PyTorch2.5.1. Optimization was performed using AdamW with a learning rate of 3 × 10^−4^ and a weight decay of 1 × 10^−4^. The batch size was 32 for the annual data and 64 for the daily data. The final models were trained for up to 10 epochs with patience-based early stopping, and the best checkpoint was selected according to the validation self-supervised loss in Equation (6). On the workstation used in this study (NVIDIA RTX 3060 Ti GPU), the complete training and inference workflow required approximately 3 min for each data set.

### 3.2. Comparison Methods

Three denoising methods were used as references: wavelet transform, Kalman filtering, and BiLSTM-CNN. These baselines were chosen because they cover threshold-based signal processing, recursive state-space smoothing, and the published learning-based benchmark developed for the same monitoring scenario. Supervised modern denoisers such as DnCNN or fully supervised Transformer denoisers were not included as direct baselines because they require clean or paired training targets, which are unavailable for the present BOTDR field measurements. Instead, the contribution of the proposed self-supervised components is examined through the component-level sensitivity analysis reported in Section 4.3.

(1) Wavelet transform

For a discrete strain signal x[n], the wavelet coefficients $W_{j,k}$ were obtained through the discrete wavelet transform in Equation (7):

(7) $$W_{j,k}=\sum_{n}x[n]\psi_{j,k}[n]$$

where $\psi_{j,k}[n]$ denotes the scaled and translated mother wavelet at decomposition level *j* and translation index *k*. After decomposition, soft thresholding was applied to the detail coefficients according to Equation (8):

(8) $${\overset{\sim }{W}}_{j,k}=sgn(W_{j,k})max\left(|W_{j,k}|-\tau ,0\right)$$

with the universal threshold in Equation (9):

(9) $$\tau =\sigma \sqrt{2logN},\sigma =\frac{MAD(W_{J})}{0.6745}$$

where *N* is the signal length and $W_{J}$ represents the highest-frequency detail coefficients. The denoised signal is reconstructed from the thresholded wavelet coefficients as in Equation (10):

(10) $$\hat{y}[n]=A_{J}[n]+\sum_{j=1}^{J}{\overset{\sim }{D}}_{j}[n]$$

where the approximation component at the coarsest level is reconstructed together with the thresholded detail components. In the present implementation, the sym8 wavelet with up to eight decomposition levels was used, consistent with the wavelet-processing strategy reported by Wei et al. [2]. The method is retained because wavelet thresholding remains one of the most common tools for suppressing localized high-frequency noise in distributed strain measurements, although its fixed threshold mechanism can also attenuate narrow peaks and valleys that may still be mechanically meaningful.

(2) Kalman filtering

The Kalman baseline was formulated as a first-order linear state-space model in Equation (11),

(11) $$s_{k}=As_{k-1}+w_{k},z_{k}=Hs_{k}+v_{k}$$

where $s_{k}$ is the latent state vector at sampling index k, $z_{k}$ is the observed strain value, A is the state transition matrix, H is the observation matrix, $w_{k}$ is the process-noise vector, and $v_{k}$ is the observation noise. The prediction step is written as Equation (12):

(12) $${\hat{s}}_{k|k-1}=A{\hat{s}}_{k-1|k-1},P_{k|k-1}=AP_{k-1|k-1}A^{T}+Q$$

where ${\hat{s}}_{k|k-1}$ is the predicted state estimate at sampling index k, ${\hat{s}}_{k-1|k-1}$ is the updated state estimate at sampling index k−1, $P_{k|k-1}$ is the predicted error covariance matrix, $P_{k-1|k-1}$ is the updated error covariance matrix at sampling index k−1, and Q is the process-noise covariance matrix and the update step is written as Equations (13)–(15):

(13) $$K_{k}=P_{k|k-1}H^{T}(HP_{k|k-1}H^{T}+R{)}^{-1}$$

(14) $${\hat{s}}_{k|k}={\hat{s}}_{k|k-1}+K_{k}\left(z_{k}-H{\hat{s}}_{k|k-1}\right)$$

(15) $$P_{k|k}=(I-K_{k}H)P_{k|k-1}$$

where $K_{k}$ is the Kalman gain matrix, R is the observation-noise covariance matrix, ${\hat{s}}_{k|k\;}$ is the updated state estimate, $P_{k|k}$ is the updated error covariance matrix, and I is the identity matrix. Consistent with Wei et al. [2], a random-walk configuration was adopted for the present comparison. The Kalman filter is retained because it provides a transparent linear recursive smoother and is widely used in engineering monitoring, but its performance is known to depend strongly on how well the assumed state evolution matches the actual strain process.

(3) BiLSTM-CNN

The BiLSTM-CNN model reported by Wei et al. [2] serves here as the principal learning-based baseline. That architecture was developed specifically for flexible-mattress strain denoising without clean labels and follows a sequence-regression strategy rather than a blind-spot formulation. In the reproduced configuration used here, the measured strain sequence is standardized and converted into overlapping spatial samples in Equation (16):

(16) $$X_{i}=[x_{i},x_{i+1},\ldots ,x_{i+L-1}],y_{i}=x_{i+L}$$

where $X_{i}$ denotes the input window starting from spatial sampling index i, $x_{i}$ is the standardized strain value at position i, L is the window length, and $y_{i}$ is the target strain value at position i+L. In this study, L was set to 20. The network therefore learns to regress the next spatial sample from the preceding 20-point neighborhood. The predicted value is assigned to the target position *i* + *L* along the monitored cable; positions without a valid preceding window are not used when reconstructing the predicted segment. Architecturally, the model contains a first bidirectional LSTM layer, dropout regularization, a one-dimensional convolutional block for local feature extraction, max pooling, a second BiLSTM layer, and a final dense regression head. In the reproduced setting, the first BiLSTM layer uses 100 hidden units, the dropout rate is 30%, the Conv1D layer uses 64 filters with kernel size 3, the max-pooling size is 2, and the second BiLSTM layer uses 50 hidden units.

At the recurrent level, each LSTM direction updates its gates according to Equations (17)–(19):

(17) $$f_{t}=\sigma \left(W_{f}[h_{t-1},u_{t}]+b_{f}\right),i_{t}=\sigma \left(W_{i}[h_{t-1},u_{t}]+b_{i}\right)$$

(18) $$o_{t}=\sigma \left(W_{o}[h_{t-1},u_{t}]+b_{o}\right),{\overset{\sim }{c}}_{t}=tanh\left(W_{c}[h_{t-1},u_{t}]+b_{c}\right)$$

(19) $$c_{t}=f_{t}⊙c_{t-1}+i_{t}⊙{\overset{\sim }{c}}_{t},h_{t}=o_{t}⊙tanh(c_{t})$$

where $u_{t}$ is the input feature vector at recurrent step t; $f_{t}$, $i_{t}$, and $o_{t}$ are the forget gate, input gate, and output gate, respectively; ${\tilde{c}}_{t}$ is the candidate cell state; $c_{t}$ is the cell state; $h_{t}$ is the hidden state; σ(⋅) is the sigmoid activation function; tanh(⋅) is the hyperbolic tangent activation function; and ⊙ denotes element-wise multiplication.

The bidirectional representation then concatenates the forward and backward hidden states as Equation (20),

(20) $$h_{t}^{bi}=\left[\overset{\to }{h_{t}};\overset{\leftarrow }{h_{t}}\right]$$

after which convolutional feature extraction is performed on the stacked hidden representation and mapped to the predicted strain by Equation (21):

(21) $$z_{t}=\sigma \left(W_{c}*h_{t}^{bi}+b_{c}\right),{\hat{y}}_{t}=W_{o}z_{t}+b_{o}$$

where ∗  denotes the one-dimensional convolution operation, $z_{t}$ is the extracted convolutional feature representation, and ${\hat{y}}_{t}$ is the predicted strain value.

Training follows a mean-squared-error objective given by Equation (22):

(22) $$L_{MSE}=\frac{1}{N}\sum_{t=1}^{N}{\left({\hat{y}}_{t}-y_{t}\right)}^{2}$$

where N is the number of training samples and $y_{t}$ is the target strain value corresponding to ${\hat{y}}_{t}$. The model was optimized with Adam at a learning rate of 1 × 10^−4^, batch size 16, and up to 1000 epochs with an internal validation split of 0.2. The raw one-step predictions are first placed in target-position order to form a one-dimensional predicted sequence. The final BiLSTM-CNN curve is then obtained by applying EWMA with span 20 point-wise to this predicted sequence, followed by a trailing 10-point moving average, as shown in Equation (23):

(23) $${\overset{\sim }{y}}_{i}={MA}_{10}\left({EWMA}_{20}({\hat{y}}_{i})\right)$$

**Figure 6:**
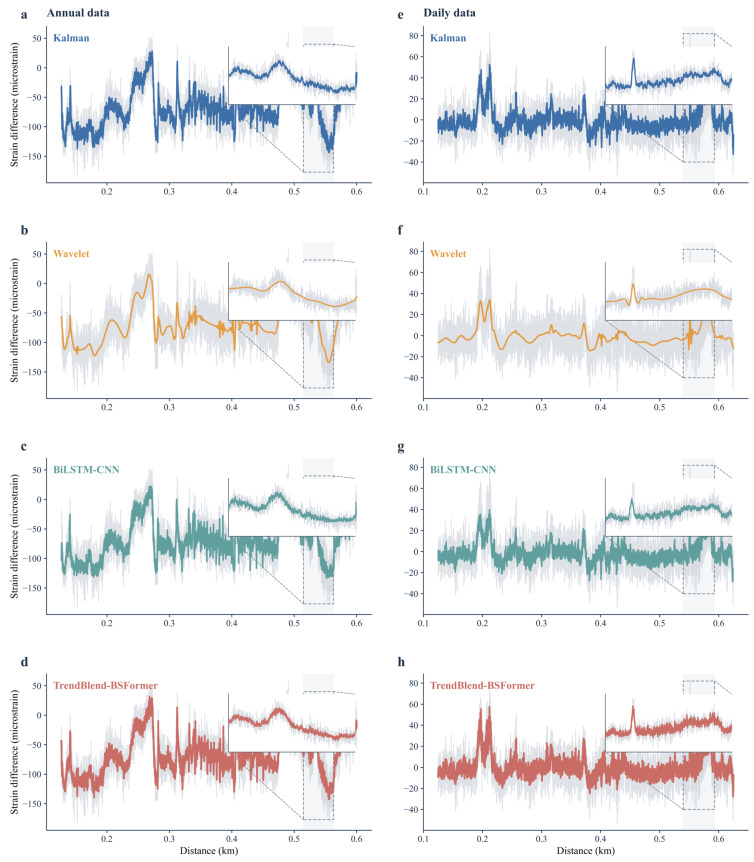
Method-specific comparison of denoised BOTDR strain-difference curves (colored lines) and raw measurements (grey lines): (**a**) annual and (**b**) daily data sets for Kalman filtering; (**c**) annual and (**d**) daily data sets for Wavelet transform; (**e**) annual and (**f**) daily data sets for BiLSTM-CNN; (**g**) annual and (**h**) daily data sets for TrendBlend-BSFormer.

The published pseudo-SNR, RMSE, and MAE values reported by Wei et al. [2] are strictly adopted as the golden quantitative benchmarks in Table 2 to preserve an unbiased comparison protocol. The locally reproduced model, executed by duplicating the released algorithmic framework as closely as possible, is utilized solely to synthesize the continuous spatial trajectories in Figure 6 for visual profile morphology inspection.

It is structurally critical to note that absolute mathematical equivalence between the original implementation and the reproduced curve is bounded by two inherent constraints. First, deep-learning training dynamics exhibit a minor degree of stochasticity under non-deterministic GPU floating-point operations. Second, due to the autoregressive sliding-window design formulated in Equation (16), the benchmark framework suffers from a cold-start constraint, rendering it incapable of generating denoised outputs for the first *L* = 20 boundary positions along the U-shaped monitored cable. This boundary truncation is transparently accommodated in our comparative figures to ensure a high-fidelity evaluation.

### 3.3. Evaluation Indicators

Three evaluation indicators were used to compare the methods. Their definitions are given in Equations (24)–(26). Because no clean reference strain is available, these indicators should be interpreted as consistency-based indices relative to the measured field signal, not as direct estimates of physical denoising error.

The pseudo-SNR used for same-protocol comparison is calculated by Equation (24).

(24) $$SNR=10{log}_{10}\left(\frac{\sum_{i=1}^{n}x_{i}^{2}}{\sum_{i=1}^{n}(x_{i}-{\hat{y}}_{i}{)}^{2}}\right)$$

where *x_i_* is the measured noisy strain and ${\hat{y}}_{i}$ is the denoised output. This quantity is therefore a pseudo-SNR defined with respect to the measured BOTDR sequence rather than a physical SNR relative to an unknown clean strain field.

Root mean square error (RMSE) is defined by Equation (25).

(25) $$RMSE=\sqrt{\frac{1}{n}\sum_{i=1}^{n}(x_{i}-{\hat{y}}_{i}{)}^{2}}$$

Mean absolute error (MAE) is defined by Equation (26).

(26) $$MAE=\frac{1}{n}\sum_{i=1}^{n}|x_{i}-{\hat{y}}_{i}|$$

No clean ground truth is available, so Equations (24)–(26) quantify consistency with the measured field signal rather than absolute recovery of the unknown true strain. This distinction is important in Brillouin sensing, where aggressive denoising may improve apparent smoothness but not necessarily produce a commensurate gain in physical measurement reliability [14]. Therefore, the numerical indicators in this paper are interpreted together with curve morphology, spectral content, and spatial-continuity evidence.

Furthermore, in real-world underwater flexible mattress monitoring, relying solely on standard error metrics (e.g., MAE or RMSE) calculated against the raw noisy reference can be mathematically deceptive. Because in situ monitoring lacks absolute ground-truth signals, an over-faithful baseline might simply overfit the non-physical high-frequency artifacts. To bridge the gap between statistical evaluation and geo-mechanical interpretability, we propose a multi-dimensional physical verification paradigm. Specifically, the spatial gradient standard deviation (δ∇) is calculated along the cable to evaluate structural boundary continuity, and the high-frequency energy ratio ($\gamma_{HF}$) is extracted via spatial Fourier transform to quantify the suppression of coherent instrumentation jitter. This dual-validation framework ensures that the denoising performance aligns with the continuous mechanical deformation characteristics of the subgrade-mattress structure.

## 4. Results and Discussion

### 4.1. Visual Comparison of Denoised Strain-Difference Curves

The denoising results are presented in Figure 6. In the absence of a clean reference field, visual inspection remains an indispensable complement to quantitative evaluation. This limitation is particularly critical in Brillouin-based distributed sensing, where denoising may improve apparent smoothness and local correlation without necessarily producing a commensurate gain in the physical reliability of the measurements [14]. In the present study, the annual data are characterized primarily by broad and persistent spatial variations, whereas the daily data exhibit much denser short-range oscillations and substantially stronger high-frequency content, consistent with the higher spectral entropy and high-frequency energy ratio reported in Section 2. These contrasts are also physically meaningful from the perspectives of river dynamics and mattress mechanics: annual strain differences are more likely to reflect cumulative bed readjustment, settlement, and sediment redistribution under seasonal hydrodynamic forcing, whereas daily differences represent a much weaker deformation signal superimposed on cable-environment interactions, temperature sensitivity, and instrumental noise. A denoising method that is credible across both scenarios must therefore suppress incoherent spikes while preserving the spatial morphology of the underlying deformation response.

From this perspective, TrendBlend-BSFormer performs more convincingly than the comparison methods. For the annual series, it suppresses isolated spikes more effectively than Kalman filtering while preserving local undulations more faithfully than wavelet thresholding, which tends to flatten the profile once smoothing becomes dominant. Compared with the BiLSTM-CNN benchmark, the proposed model captures the overall deformation trend more coherently and retains peaks and troughs that remain mechanically plausible for a submerged flexible mattress responding to distributed support loss and local scour. This distinction is not merely visual. Flexible mattresses act as deformable protective layers rather than rigid structural members; accordingly, their strain response should generally exhibit a degree of spatial continuity along the protected bed, except in zones affected by localized instability or abrupt support changes [1,2]. The contrast is even more evident for the daily series, in which the proposed model attenuates high-frequency jitter without eliminating short-range fluctuations that may still correspond to localized disturbances or abrupt changes in cable response. A similar emphasis on preserving coherent signal structures, rather than simply minimizing noise amplitude, has also been highlighted in recent self-supervised denoising studies of distributed sensing data [22,23].

### 4.2. Quantitative Performance Comparison

**Figure 7:**
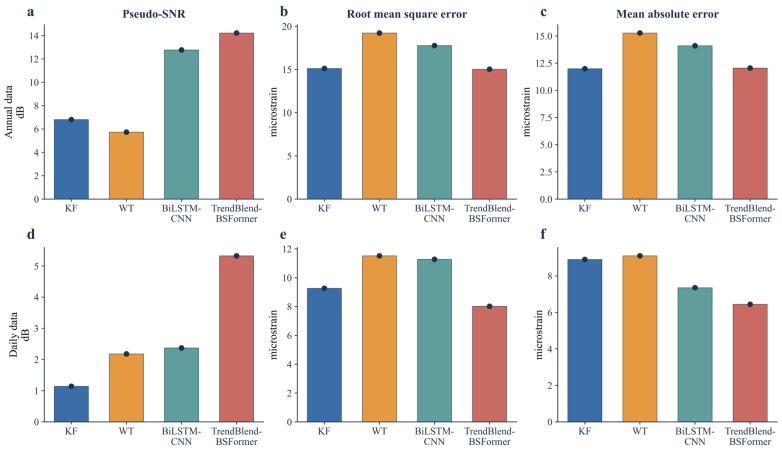
Quantitative comparison of pseudo-SNR, RMSE, and MAE for the annual and daily data sets: (**a**–**c**) annual pseudo-SNR, RMSE, and MAE; (**d**–**f**) daily pseudo-SNR, RMSE, and MAE.

The quantitative comparison in Figure 7 and Table 2 corroborates the visual assessment. For the annual data, TrendBlend-BSFormer improves the pseudo-SNR by 1.45 dB relative to the BiLSTM-CNN benchmark reported by Wei et al. [2], while reducing the RMSE and MAE by 2.74 and 2.05 με, respectively. For the daily data, the relative advantage is more pronounced, with a pseudo-SNR increase of 2.95 dB, an RMSE reduction of 3.26 με, and an MAE reduction of 0.91 με. The absolute pseudo-SNR of TrendBlend-BSFormer is higher for the annual data (14.22 dB) than for the daily data (5.32 dB), because the annual sequence contains a larger coherent deformation component and broader spatial persistence. The daily data have a weaker mean deformation signal and substantially richer high-frequency content; therefore, their absolute pseudo-SNR remains lower, although the gain relative to the baseline methods is larger. This distinction was added to avoid confusing absolute pseudo-SNR with relative improvement.

When compared across methods, the quantitative results also clarify the practical trade-offs involved. It is noteworthy in Table 2 that the first-order Kalman filter achieves a marginally lower MAE (11.99 με) on the annual dataset compared to TrendBlend-BSFormer (12.05 με). However, as analyzed from the multi-dimensional physical validation perspective, this minor numerical superiority is an artifact of over-fitting rather than superior structural recovery. The Kalman filter recursively minimizes variance by adhering too strictly to the raw measured sequence, thereby inadvertently preserving the non-physical isolated spikes caused by weak spontaneous Brillouin scattering in the BOTDR sensing chain. In riverway engineering practice, retaining these single-point instrumental fluctuations is highly problematic, as it would generate frequent false alarms for structural failure.

In contrast, TrendBlend-BSFormer dramatically reduces the gradient standard deviation from 24.27 to 5.14 με and suppresses the annual high-frequency energy ratio to 0.011. By selectively filtering out these uncoordinated point-wise spikes while maintaining a smooth macro-trend, our framework trades a negligible drop in raw noise-fitting proximity for a substantial gain in geo-mechanical plausibility, successfully separating systemic optical jitter from authentic, continuous riverbed morphology variations.

### 4.3. Interpretation of Indicator Response and Denoising Behavior

The indicator behavior must be interpreted together with structural descriptors. For the annual data, the high-frequency energy ratio decreases from 0.172 for the raw sequence to 0.011 for TrendBlend-BSFormer, while the gradient standard deviation decreases from 24.27 to 5.14 με per point. For the daily data, the corresponding high-frequency energy ratio decreases from 0.424 to 0.112, and the gradient standard deviation decreases from 15.18 to 6.01 με per point. These values show that the proposed model suppresses a large portion of incoherent high-frequency fluctuation while retaining more short-range structure than overly smooth wavelet outputs. Thus, the slightly higher annual MAE relative to Kalman filtering should not be read as inferior physical recovery; rather, it reflects the limitation of evaluating denoising against the noisy measurement itself. As discussed by Soto, Ramirez [14], improvements in apparent pseudo-SNR after denoising do not automatically translate into equivalent gains in Brillouin-frequency uncertainty or physical measurement fidelity.

The behavior of TrendBlend-BSFormer is better understood in the context of recent advances in blind and self-supervised denoising. Methods based on J-invariance and blind-spot masking exploit redundancy within the observation itself, allowing coherent structures to be reconstructed from neighboring information without requiring clean targets [20,21]. In distributed sensing, related strategies have been shown to improve waveform coherence and suppress spatially incoherent noise even when the useful signal is weaker than the surrounding disturbance [22,23,24,25]. The present model extends this logic to BOTDR strain-difference data through the combined action of Equations (1)–(6). The component-level sensitivity results in Table 3 further show that the Transformer bottleneck, cross-view consistency, and residual-noise head play different roles: the Transformer contributes mainly to long-range spatial coherence, the consistency term stabilizes blind-mask predictions, and the noise head makes the residual representation explicit.

### 4.4. Limitations and Implications for Field Interpretation

These results should nevertheless be interpreted with appropriate caution. The validation uses one monitoring site, one type of underwater protection structure, and two strain-difference data sets derived from the same BOTDR system. The annual and daily models were trained separately because the two signal classes differ substantially in amplitude and dominant scale; whether a single unified model can generalize across multiple monitoring intervals, hydraulic conditions, cable types, and structural settings remains an open question. Moreover, the trend kernels and the hyperparameters gamma, lambda, and eta are empirical. A sensitivity scan for the annual data showed that increasing detail preservation improved the consistency-based indicators (pseudo-SNR varied from 14.04 to 15.63 dB across tested settings) but also increased curve roughness, with gradient standard deviation rising from 4.32 to 10.22 με per point. The selected configuration was therefore chosen as a compromise between numerical consistency and visually interpretable curve morphology, rather than as a claim of global optimality. Future work should test the framework on additional sites and include physics-informed constraints, uncertainty quantification, and external validation against independent settlement or bathymetric observations.

Even with these limitations, the present findings have clear implications for field applications. The strain measured on an underwater flexible mattress represents an integrated response of the sensing cable, the mattress body, and the supporting riverbed; accordingly, its engineering significance depends on whether the distributed profile can still be interpreted as a plausible deformation pattern after denoising. A method that preserves mechanically credible curve morphology while reducing incoherent fluctuations can make multi-period comparisons more defensible, help distinguish localized anomalies from measurement artefacts, and improve the readability of distributed strain profiles for engineering judgment. In this sense, TrendBlend-BSFormer should be understood not as a purely cosmetic post-processing tool, but as a method that helps bridge the gap between raw BOTDR acquisition and interpretable evidence for assessing the service condition of underwater protection systems.

## 5. Conclusions

This study developed TrendBlend-BSFormer, a blind self-supervised denoising framework for in situ BOTDR strain-difference data acquired from underwater flexible mattresses in the absence of clean reference signals. The proposed framework combines a one-dimensional encoder–decoder backbone, a Transformer bottleneck, and a multi-scale trend-detail blending branch, thereby enabling direct learning from noisy field measurements while preserving the curve morphology essential for engineering interpretation.

Using annual and daily BOTDR datasets collected from the Yudaizhou shoreline protection project, the proposed method outperformed wavelet transform, Kalman filtering, and the published BiLSTM-CNN benchmark under the same indicator protocol. Specifically, it achieved pseudo-SNR, RMSE, and MAE values of 14.22 dB, 15.03 με, and 12.05 με, respectively, for the annual dataset, and 5.32 dB, 8.02 με, and 6.45 με, respectively, for the daily dataset. The comparison with BiLSTM-CNN is based on the published benchmark values and a reproduced target-aligned curve generated following the released implementation; this distinction is now explicitly stated in the revised manuscript.

These findings demonstrate that denoising is a fundamental prerequisite for translating BOTDR strain measurements into reliable engineering interpretation, rather than merely serving as a smoothing procedure. The revised analysis also clarifies the limits of the present validation: the reported pseudo-SNR/RMSE/MAE values are consistency indices relative to noisy field measurements, the available data come from a single monitoring project, and the trend parameters remain empirical. Even with these limitations, the results indicate that blind self-supervised denoising offers a promising route toward more readable and physically plausible distributed-strain profiles for underwater protection systems.

## Figures and Tables

**Table 1:** Monitoring and statistical descriptors of the annual and daily BOTDR strain-difference data sets.

Descriptor	Annual Data Set	Daily Data Set
Period used for strain difference	18 Nov. 2020 to 28 Nov. 2021	18 Nov. 2020 to 19 Nov. 2020
Valid measurement points	9343	9875
Approximate sampling interval	0.05 m	0.05 m
Mean	−67.74 με	−0.11 με
Standard deviation	37.20 με	14.80 με
Minimum	−183 με	−53 με
Maximum	58 με	82 με
Median	−70 με	−2 με
Interquartile range	51 με	18 με
Median absolute deviation	25 με	9 με
Skewness	0.24	0.79
Excess kurtosis	−0.24	1.68
Lag-1 autocorrelation	0.787	0.474
Mean absolute first difference	19.30 με	12.00 με
Gradient standard deviation	24.27 με per point	15.18 με per point
Spectral entropy	0.551	0.784
High-frequency energy ratio	0.172	0.424

**Table 2:** Quantitative comparison of the denoising methods for the annual and daily data sets.

Data Set	Method	Pseudo-SNR (dB)	RMSE (Με)	MAE (Με)
Annual	Kalman filtering	6.81	15.12	11.99
Annual	Wavelet transform	5.73	19.22	15.27
Annual	BiLSTM-CNN [2]	12.77	17.77	14.10
Annual	TrendBlend-BSFormer	14.22	15.03	12.05
Daily	Kalman filtering	1.14	9.27	8.90
Daily	Wavelet transform	2.18	11.52	9.10
Daily	BiLSTM-CNN [2]	2.37	11.28	7.35
Daily	TrendBlend-BSFormer	5.32	8.02	6.45

**Table 3:** Component-level sensitivity of TrendBlend-BSFormer based on self-supervised test loss.

Configuration	Annual Self-Supervised Loss	Daily Self-Supervised Loss	Interpretation
Full TrendBlend-BSFormer	0.171	0.346	Selected configuration; balances trend continuity, detail preservation, and explicit signal-noise separation.
Without Transformer bottleneck	0.176	0.357	Higher self-supervised loss, especially for the daily data; long-range spatial context becomes weaker.
Without cross-view consistency	0.169	0.345	Numerically similar but lacks the constraint that stabilizes outputs under different blind masks.
Without noise head	0.171	0.346	Numerically close to the full model; retained to keep the residual-noise representation explicit.

## Data Availability

The data are available from the corresponding author upon reasonable request.
